# Trajectories of sleep disturbances in the subacute phase after stroke: a longitudinal actigraphy study

**DOI:** 10.1007/s11325-026-03810-z

**Published:** 2026-09-16

**Authors:** Ruani Araújo Tenório, Isadora Grade Biasibetti, Maria Tereza Mota Alvarenga, Luci Fuscaldi Teixeira-Salmela, Patrick Roberto Avelino, Andressa Silva, Aline Alvim Scianni

**Affiliations:** 1 Department of Physical Therapy, Universidade Federal de Minas Gerais (UFMG), Belo Horizonte, Minas Gerais Brazil; 2 Department of Sports, Universidade Federal de Minas Gerais (UFMG), Belo Horizonte, Minas Gerais Brazil

**Keywords:** Stroke, Sleep, Actigraphy, Sleep efficiency, Longitudinal study, Sleep fragmentation

## Abstract

**Background:**

Sleep disturbances are common after stroke and may negatively impact recovery and quality of life. However, monitoring sleep parameters over time with objective measures remains underreported.

**Objective:**

To investigate longitudinal changes in actigraphy-derived sleep parameters from the subacute to later stages after stroke.

**Methods:**

This longitudinal observational study included 18 individuals with stroke who completed seven-day wrist-actigraphy monitoring at baseline and at the most distal valid follow-up assessment obtained more than three months after stroke. The median interval between assessments was 161 days (IQR 102.3–194.0). Time in bed, total sleep time, sleep latency, sleep efficiency, wake after sleep onset (WASO), and number of awakenings were compared using paired t-tests. Mean paired differences, 95% confidence intervals (CIs), and Cohen’s d effect sizes were calculated.

**Results:**

No statistically significant longitudinal changes were detected. Mean changes were − 0.12 h for time in bed (95% CI − 0.73 to 0.49), − 0.05 h for total sleep time (95% CI − 0.59 to 0.50), − 7.93 min for sleep latency (95% CI − 19.55 to 3.69), − 0.36% points for sleep efficiency (95% CI − 4.19 to 3.48), 3.63 min for WASO (95% CI − 9.22 to 16.48), and 0.43 for number of awakenings (95% CI − 0.74 to 1.59). Effect sizes were small, and exploratory individual-level analysis showed increased sleep efficiency in 10 participants and decreased sleep efficiency in 8.

**Conclusion:**

The analysis showed no statistically significant changes in sleep patterns between the subacute phase and the follow-up. While sleep metrics remained outside normal ranges at both time points, the small and heterogeneous sample, along with wide confidence intervals, prevents any definitive conclusions about long-term trends or causes.

## Introduction

Stroke remains one of the leading causes of death and long-term disability worldwide, representing a major public health burden with substantial social and economic consequences [1, 2]. Although advances in acute stroke management have improved survival rates, a large proportion of stroke survivors experience persistent neurological and systemic complications that may compromise functional recovery and quality of life. Among these complications, sleep disturbances have increasingly been recognized as an important yet often underdiagnosed component of post-stroke morbidity.

Sleep plays a fundamental role in neurological recovery, brain plasticity, and overall health. After a stroke, disturbances such as insomnia symptoms, fragmented sleep, and excessive daytime sleepiness are highly prevalent and may negatively affect cognitive recovery, mood, functional performance, and participation in daily activities [3]. Growing evidence also suggests that sleep disorders following stroke are associated with poorer functional outcomes and increased symptom burden, reinforcing the importance of systematically assessing sleep in stroke populations [4].

Despite this growing recognition, most studies investigating sleep after stroke have relied primarily on subjective measures, including questionnaires and self-reported sleep complaints. While these instruments provide important information regarding patients’ perceptions of sleep, they may not accurately reflect objective sleep–wake patterns. Objective methods are therefore essential to better characterize sleep disturbances in this population.

Actigraphy has emerged as a practical and noninvasive method to objectively assess sleep–wake patterns over several consecutive days in naturalistic environments. This technique allows the estimation of key sleep parameters, including total sleep time, sleep efficiency, sleep latency, wake after sleep onset, and sleep fragmentation, as well as the evaluation of night-to-night variability in sleep patterns. Previous studies using actigraphy have demonstrated that stroke survivors frequently exhibit reduced sleep efficiency, fragmented sleep, and altered sleep–wake patterns compared with healthy individuals [5, 6]. Additionally, actigraphy has proven to be a feasible tool for monitoring sleep and activity patterns in neurological populations under real-life conditions [7].

However, despite the increasing use of actigraphy in sleep research, the longitudinal follow-up of objectively measured sleep parameters after a stroke remains poorly understood. Most available studies are cross-sectional or focus on acute stages of stroke, limiting our understanding of how sleep patterns evolve during the recovery process. The contribution of the present study lies in the repeated assessment of sleep under naturalistic conditions using seven-day actigraphy, providing objective within-participant data across two stages of post-stroke recovery.

Therefore, this study aimed to compare actigraphy-derived sleep parameters between a subacute post-stroke assessment and a later follow-up assessment. A secondary exploratory objective was to describe individual-level changes in sleep efficiency.

## Methods

### Study design

This longitudinal observational study investigated changes in objectively measured sleep parameters in individuals after stroke. Participants were assessed at two time points during stroke recovery: the subacute phase (between 1 and 3 months after stroke) and a follow-up assessment conducted at least 3 months after the stroke. Sleep–wake patterns were evaluated using wrist actigraphy over seven consecutive days at each assessment.

### Participants

**Fig. 1:**
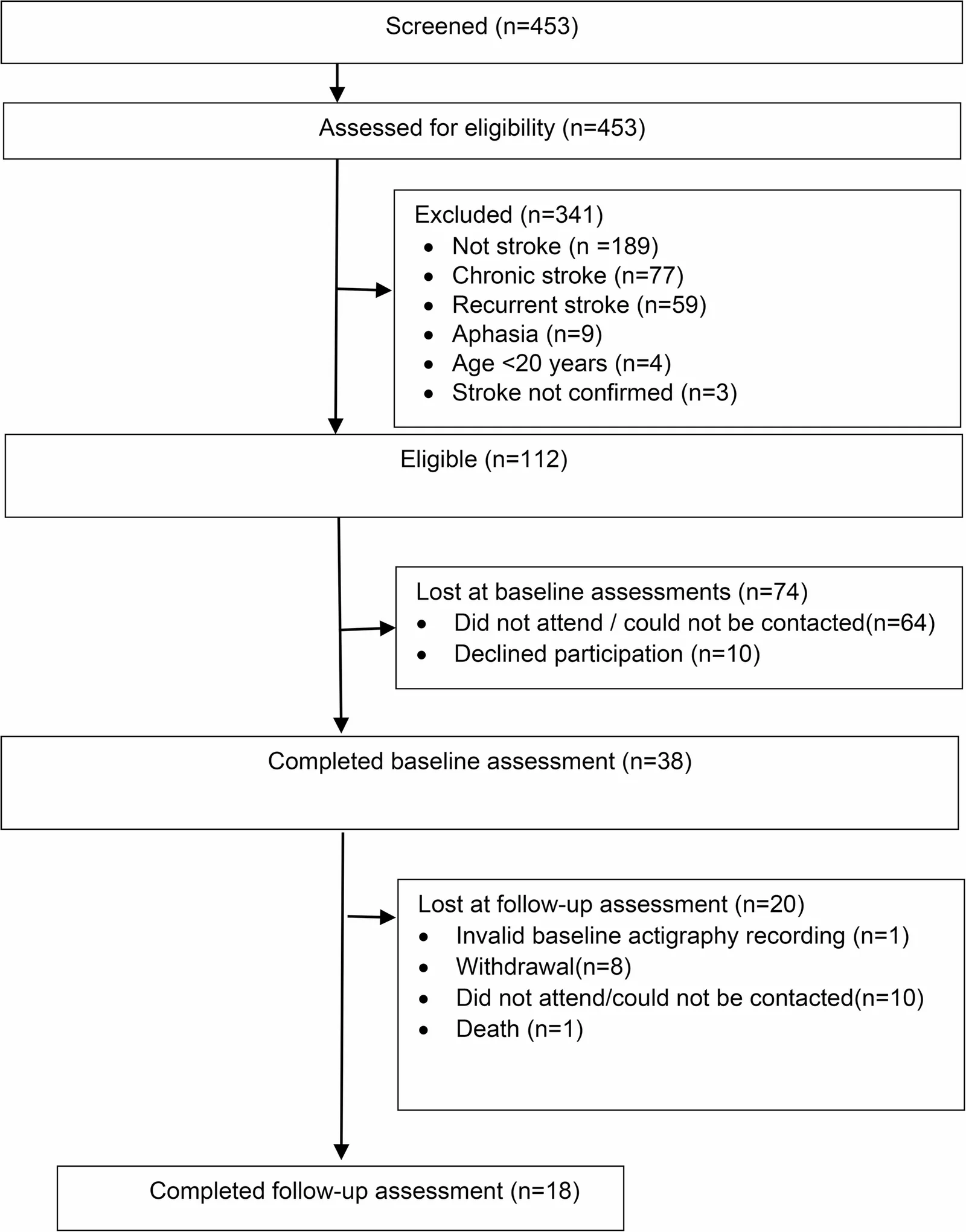
Flow of participants through the study

Participants were recruited from the Stroke Unit of Hospital Risoleta Tolentino Neves, in Brazil. Patients admitted to the Stroke Unit during the recruitment period were screened for eligibility. Repeated screening encounters were identified using the medical record number or, when this was unavailable, the participant’s normalized full name. The numbers screened, assessed for eligibility, excluded, enrolled, lost during follow-up, and included in the final paired analysis are presented in Fig. 1.

Eligible participants were adults aged ≥ 20 years with a clinical diagnosis of stroke at least four weeks prior to enrollment. Individuals were eligible if they had a confirmed diagnosis of stroke and were within the subacute phase of recovery at the time of the first assessment. For screening purposes, chronic stroke was defined as a stroke event that had occurred earlier than this subacute window (i.e., more than three months before the planned baseline assessment), and recurrent stroke was defined as a new cerebrovascular event occurring in a patient with a previously documented stroke; both conditions were treated as screening-level ineligibility criteria because they did not correspond to a single, dateable subacute stroke event, which was required to standardize the timing of assessments relative to stroke onset.

Additional inclusion criteria included preserved cognitive function sufficient to comprehend and respond to questionnaire items, as assessed by the Mini-Mental State Examination (MMSE). Cognitive eligibility followed education-adjusted cutoff scores proposed by Bertolucci et al. (1994), defined as ≥ 13 for illiterate individuals, ≥ 18 for those with 1–8 years of formal education, and ≥ 26 for participants with more than 8 years of schooling [8].

Exclusion criteria comprised: (1) moderate-to-severe cognitive impairment, indicated by MMSE scores below the education-adjusted cutoffs; (2) aphasia sufficient to compromise reliable comprehension and completion of the self-reported outcome measures and the informed consent process used in this study, irrespective of the individual’s physical capacity to wear the actigraphy device; (3) unstable medical or psychiatric conditions that could compromise participation or influence sleep patterns; and (4) occurrence of any acute clinical event between the baseline and follow-up assessments that could substantially alter sleep behavior. Demographic and clinical variables—including age, sex, stroke subtype, time since stroke, and functional characteristics—were collected to characterize the sample. Participant recruitment and follow-up are summarized in Fig. 1. Individuals were included in the longitudinal analysis only when they had a valid baseline actigraphy recording and at least one valid follow-up recording obtained more than three months after stroke.

### Procedures

Participants underwent actigraphy monitoring with ActTrust 2 (Condor Instruments^®^, São Paulo, SP) for seven consecutive days during each assessment period. The first assessment occurred between 1 and 3 months after stroke, corresponding to the subacute phase of stroke recovery. The follow-up assessment occurred at least 3 months after the stroke, representing the later stage of recovery.

Participants were instructed to wear the actigraphy device continuously on the non-paretic wrist during the monitoring period and to maintain their usual daily routines. Devices were removed only when necessary (e.g., bathing).

Sleep–wake data were recorded continuously throughout the monitoring period and subsequently processed using the manufacturer’s software in accordance with standard actigraphy scoring procedures. Participants received usual post-stroke clinical care outside the study. The type, frequency, and duration of rehabilitation received between assessments were not systematically recorded. No sleep-specific intervention was delivered as part of this observational study.

### Actigraphy measures

Sleep–wake parameters were derived from actigraphy recordings averaged across the seven monitoring nights for each assessment. The following variables were analyzed:

- Time in bed (minutes): total time spent in bed between bedtime and final awakening.
- Total sleep time (minutes): total amount of time scored as sleep during the nocturnal sleep period. Values below 6 h are generally considered indicative of short sleep duration in adults.
- Sleep latency (minutes): time required to transition from wakefulness to sleep after going to bed. Sleep latency values greater than 30 min are commonly interpreted as prolonged sleep onset.
- Sleep efficiency (%): proportion of time spent asleep while in bed. Sleep efficiency below 85% is typically considered indicative of reduced sleep quality.
- Wake after sleep onset (WASO, minutes): total duration of wakefulness occurring after sleep onset. WASO values greater than 30 min suggest increased sleep fragmentation.
- Number of awakenings: total number of nocturnal awakenings during the sleep period. Higher frequencies of awakenings reflect greater sleep fragmentation.

These parameters were selected to characterize sleep duration, sleep continuity, and sleep fragmentation. Reference values used to contextualize sleep quality were based on established normative sleep parameters described in adult populations [9].

### Clinical and demographic measures

Clinical and demographic information was collected at baseline. The following standardized clinical measures were obtained:

- Functional disability: assessed using the Modified Rankin Scale (mRS), a widely used measure of global disability and dependence in activities of daily living after stroke [10].
- Fatigue: assessed using the Fatigue Severity Scale (FSS), a self-reported instrument designed to evaluate the impact of fatigue on daily functioning and widely used in neurological populations [11].
- Depressive symptoms: assessed using the 15-item Geriatric Depression Scale (GDS-15), a screening instrument commonly used to identify depressive symptoms in older adults and validated for use in Brazilian populations [12].
- Quality of life: assessed using the EuroQol-5D-3 L visual analogue scale (EQ-VAS), which measures self-perceived health status on a scale ranging from 0 (worst imaginable health state) to 100 (best imaginable health state) [13].
- Participation: assessed using the participation domain of the Stroke Impact Scale version 3.0 (SIS 3.0), which evaluates perceived limitations in social participation after stroke [14].

All instruments used have demonstrated adequate measurement properties in neurological populations [10–14].

These measures were used to characterize the clinical and functional profile of the participants.

### Statistical analysis

Descriptive statistics were used to summarize demographic, clinical, and sleep variables. Continuous variables were presented as mean (SD) when normally distributed or as median and interquartile range (IQR) when distributional assumptions were not met, while categorical variables were described using absolute and relative frequencies (n, %) [15].

The normality of the paired differences between follow-up and baseline was assessed separately for each actigraphy-derived parameter using the Shapiro–Wilk test. Variables with approximately normal paired differences (sleep efficiency, number of awakenings) were compared using paired t tests, whereas variables that did not meet the normality assumption (time in bed, total sleep time, sleep latency, WASO) were compared using Wilcoxon signed-rank tests. All comparisons were repeated using the alternative test (the paired t test for variables analyzed primarily with the Wilcoxon signed-rank test, and vice versa); results were consistent across approaches.

For each paired comparison, the mean difference between follow-up and baseline and its 95% confidence interval were calculated. Standardized paired effect sizes were expressed as Cohen’s d with 95% confidence intervals. Given the small and exploratory sample, emphasis was placed on effect estimates and confidence intervals rather than on statistical significance alone.

Individual-level changes in sleep efficiency were explored descriptively using the participant-level mean values across the valid monitoring nights. Change was calculated as follow-up minus baseline. Positive values were classified as increases and negative values as decreases. These classifications indicate only the direction of change and should not be interpreted as clinically meaningful improvement or deterioration because no established minimum clinically important difference is available for actigraphy-derived sleep efficiency after stroke.

All analyses were conducted using SPSS Statistics version 21.0, and all analyses were independently verified by a second researcher to ensure analytical reproducibility. Statistical significance was set at α = 0.05, and all tests were two-sided.

## Results

### Participant flow

Between November 2023 and September 2025, 453 individuals were screened for eligibility. Of these, 341 were excluded because they did not meet the eligibility criteria, including not having a stroke (*n* = 189), chronic stroke (*n* = 77), recurrent stroke (*n* = 59), aphasia (*n* = 9), age < 20 years (*n* = 4), or an unconfirmed stroke diagnosis (*n* = 3). Consequently, 112 individuals met the eligibility criteria.

Among the eligible participants, 74 did not complete the baseline assessment, including 64 who did not attend or could not be contacted and 10 who declined participation. Therefore, 38 participants completed the baseline assessment. One participant had an invalid baseline actigraphy recording and was excluded from the longitudinal analyses. Of the remaining participants, eight withdrew from the study, ten did not attend the follow-up assessment or could not be contacted, and one died during follow-up. Consequently, 18 participants completed valid actigraphy assessments at both time points and were included in the paired longitudinal analysis (Fig. 1).

### Participant characteristics

A total of 18 participants with confirmed stroke completed actigraphy assessments at both time points and were included in the longitudinal analyses. Baseline demographic and clinical characteristics are presented in Table 1.

**Table 1:** Clinical and demographic characteristics of the study participants (*n* = 18)

Variable	Value
Age (years), mean (SD)	63.0 (11.4)
Sex, n (%)	
Male	11 (61.1)
Female	7 (38.9)
Race, n (%)	
Brown/mixed (Pardo)	11 (61.1)
Black	2 (11.1)
White	1 (5.6)
Education (years) mean (SD)	8.7 (3.6)
Body mass index (kg/m²) mean (SD)	27.2 (5.9)
Time since stroke (days) mean (SD)	56.9 (19.4)
Type of stroke, n (%)	
Ischemic	16 (88.9)
Hemorrhagic	2 (11.1)
Stroke topography (Bamford), n (%)	
LACI	4 (22.2)
PACI	3 (16.7)
POCI	2 (11.1)
Stroke etiology (TOAST), n (%)	
Undetermined	6 (33.3)
Cardioembolism	4 (22.2)
Small-vessel occlusion	2 (11.1)
Hypertension, n (%)	14 (77.8)
Diabetes mellitus, n (%)	8 (44.4)
Smoking status, n (%)	
Never smoker	8 (44.4)
Former smoker	10 (55.6)
NIH Stroke Scale (NIHSS), median (IQR)	4.0 (3.0–4.0)
Mini-Mental State Examination (MMSE), median (IQR)	27.0 (24.2–28.0)
Modified Rankin Scale (mRS), median (IQR)	2.0 (1.0–3.0)
Fatigue Severity Scale (FSS), median (IQR)	39.5 (13.2–52.2)
Geriatric Depression Scale (GDS), median (IQR)	4.0 (3.0–6.0)
EuroQol-5D-3 L VAS, mean (SD)	63.2 (19.8)
SIS 3.0 – Social Participation, median (IQR)	78.1 (43.0–96.1)

Values are presented as mean (standard deviation) or median (interquartile range), as appropriate. Categorical variables are presented as n (%)

The mean age of the participants was 63.0 (SD 11.4) years, and most were male (61.1%). The majority of strokes were ischemic (88.9%), while 11.1% were hemorrhagic. Regarding vascular risk factors, hypertension was present in 77.8% of participants and diabetes mellitus in 44.4%.

Baseline neurological impairment was generally mild. The median NIH Stroke Scale (NIHSS) score was 4.0 (IQR 3.0–4.0), and the median modified Rankin Scale (mRS) score was 2.0 (IQR 1.0–3.0), indicating mild to moderate functional disability. Cognitive performance, assessed with the Mini-Mental State Examination (MMSE), had a median score of 27.0 (IQR 24.2–28.0).

Median FSS and GDS-15 scores were 39.5 (IQR 13.2–52.2) and 4.0 (IQR 3.0–6.0), respectively. The mean EQ-VAS score was 63.2 (SD 19.8), and the median SIS 3.0 participation score was 78.1 (IQR 43.0–96.1).

### Actigraphy-derived sleep parameters

Actigraphy-derived sleep parameters at baseline and follow-up are presented in Table 2. The median interval between the baseline and follow-up assessments was 161 days (IQR 102.3–194.0; range 63–391 days). Follow-up assessments were conducted a median of 216 days after stroke (IQR 160.0–237.8; range 103–465 days). The follow-up assessment corresponded to the most distal valid actigraphy recording obtained more than three months after stroke (Table 3).

**Table 2:** Actigraphy-derived sleep parameters at baseline and follow-up (*n* = 18) Baseline: 1–3 months post-stroke (AV1) Follow-up: ≥3 months post-stroke

Variable	Baseline, mean (SD)	Follow-up, mean (SD)	Mean change	95% CI	Cohen’s d	95% CI for dz	*p*-value
Time in bed, h	8.24 (1.59)	8.12 (1.86)	−0.12	−0.73 to 0.49	−0.10	−0.56 to 0.37	0.687 ^**a**^
Total sleep time, h	6.63 (1.38)	6.59 (1.83)	−0.05	−0.59 to 0.50	−0.04	−0.51 to 0.42	0.853 ^**a**^
Sleep latency, min	43.57 (28.26)	35.64 (20.73)	−7.93	−19.55 to 3.69	−0.34	−0.81 to 0.14	0.168 ^**a**^
Sleep efficiency, %	80.49 (6.54)	80.13 (8.73)	−0.36	−4.19 to 3.48	−0.05	−0.51 to 0.42	0.847 ^**b**^
WASO, min	47.30 (17.84)	50.93 (23.00)	3.63	−9.22 to 16.48	0.14	−0.33 to 0.60	0.559 ^**a**^
Number of awakenings	5.71 (2.67)	6.14 (3.08)	0.43	−0.74 to 1.59	0.18	−0.29 to 0.65	0.450 ^**b**^

Values are presented as mean (standard deviation)

Mean change was calculated as follow-up minus baseline

Paired comparisons were performed using Wilcoxon signed-rank ^a^ and paired t tests ^b^

Standardized paired effect sizes are presented as Cohen’s d

*CI* confidence interval, *WASO* wake after sleep onset

**Table 3:** Individual changes in sleep efficiency between baseline and follow-up (*n* = 18)

Participant	Follow-up (Days after stroke)	Baseline sleep efficiency (%)	Follow-up sleep efficiency (%)	Change (percentage points)	Direction
Participant 1	159	71.85	84.59	12.74	Increase
Participant 2	221	75.45	61.62	−13.83	Decrease
Participant 3	238	84.79	67.35	−17.44	Decrease
Participant 4	211	79.25	87.54	8.29	Increase
Participant 5	150	76.39	81.65	5.26	Increase
Participant 6	177	80.56	81.35	0.79	Increase
Participant 7	222	81.48	88.19	6.71	Increase
Participant 8	194	84.02	84.36	0.34	Increase
Participant 9	230	66.17	72.98	6.81	Increase
Participant 10	237	90.43	86.31	−4.12	Decrease
Participant 11	465	81.68	84.34	2.66	Increase
Participant 12	151	85.18	80.35	−4.83	Decrease
Participant 13	163	68.59	59.77	−8.82	Decrease
Participant 14	323	84.24	82.48	−1.76	Decrease
Participant 15	358	83.08	86	2.92	Increase
Participant 16	332	83.28	82.22	−1.06	Decrease
Participant 17	155	83.35	86.63	3.28	Increase
Participant 18	103	88.94	84.59	−4.35	Decrease

Change was calculated as follow-up minus baseline. Direction indicates increase or decrease only and does not imply clinically meaningful improvement or deterioration

At baseline, mean time in bed was 8.24 (SD 1.59) hours, mean total sleep time was 6.63 (SD 1.38) hours, mean sleep latency was 43.57 (SD 28.26) minutes, and mean sleep efficiency was 80.49% (SD 6.54).

Mean WASO was 47.30 (SD 17.84) minutes, and the mean number of nocturnal awakenings was 5.71 (SD 2.67). At follow-up, mean time in bed was 8.12 (SD 1.86) hours, mean total sleep time was 6.59 (SD 1.83) hours, mean sleep latency was 35.64 (SD 20.73) minutes, mean sleep efficiency was 80.13% (SD 8.73), mean WASO was 50.93 (SD 23.00) minutes, and the mean number of awakenings was 6.14 (SD 3.08).

Using commonly applied reference thresholds, total sleep time was below 7 h in 61.1% of participants at baseline and 55.6% at follow-up. Sleep latency exceeded 30 min in 72.2% and 50.0%, sleep efficiency was below 85% in 83.3% and 72.2%, and WASO exceeded 30 min in 83.3% at both assessments, respectively.

### Longitudinal comparisons

No statistically significant longitudinal changes were detected for any actigraphy-derived sleep parameter. Mean paired changes were − 0.12 h for time in bed (95% CI − 0.73 to 0.49; *p* = 0.687; Cohen’s d = − 0.10), − 0.05 h for total sleep time (95% CI − 0.59 to 0.50; *p* = 0.853; d = − 0.04), − 7.93 min for sleep latency (95% CI − 19.55 to 3.69; *p* = 0.168; d = − 0.34), − 0.36% points for sleep efficiency (95% CI − 4.19 to 3.48; *p* = 0.847; d = − 0.05), 3.63 min for WASO (95% CI − 9.22 to 16.48; *p* = 0.559; d = 0.14), and 0.43 for number of awakenings (95% CI − 0.74 to 1.59; *p* = 0.450; d = 0.18). Non-parametric sensitivity analyses yielded the same interpretation.

All standardized point estimates were small, but their confidence intervals were wide and included changes in both directions. Therefore, the non-significant findings should not be interpreted as evidence of equivalence or absence of change. In the exploratory individual-level analysis, sleep efficiency increased in 10 participants and decreased in 8 participants.

Individual sleep-efficiency data are presented in Table 3. The classifications describe only the direction of any observed change and should not be interpreted as clinically meaningful improvement or deterioration because no established minimum clinically important difference is available for this outcome after stroke.

## Discussion

The present study investigated longitudinal changes in objectively measured sleep parameters in individuals from the subacute to later stages of stroke recovery using actigraphy. No statistically significant changes were detected in actigraphy-derived sleep parameters between the subacute assessment and follow-up. Mean paired changes and standardized effect sizes were small; however, the confidence intervals were wide and included changes in both directions. The absence of significant change suggests that sleep quality may have remained relatively stable, but given the small sample and wide confidence intervals, these findings should be interpreted with caution and should not be read as evidence of equivalence between the two time points.

Sleep disturbances are increasingly recognized as an important component of post-stroke morbidity, with non-motor complications potentially affecting recovery and long-term outcomes [1, 2]. Previous studies employing subjective and objective measures have reported a high prevalence of insomnia symptoms, fragmented sleep, and excessive daytime sleepiness among stroke survivors, associated with poorer neurological outcomes, greater fatigue, and reduced quality of life [3, 4, 7, 16–18].

The persistence of altered sleep parameters observed in this study is consistent with previous objective sleep assessments after stroke, which have shown reduced sleep efficiency, increased nocturnal awakenings, fragmented sleep–wake patterns, and irregular rest–activity rhythms compared with healthy individuals or population norms [5, 6, 9]. Together, these findings suggest that sleep disturbances following stroke may persist beyond the acute phase and may represent a long-term consequence of neurological injury.

Beyond the absence of significant longitudinal changes, it is essential to consider the absolute values of the sleep parameters observed in this study, which indicate that sleep quality was already compromised in the subacute phase and remained suboptimal at follow-up. When compared with normative values for healthy adults, most parameters fell outside expected ranges at both assessments: total sleep time (~ 6.6 h) was below the recommended 7–9 h, sleep latency (~ 35.6–43.6 min) exceeded the 30-minute threshold, sleep efficiency (~ 80%) was below the expected ≥ 85%, and WASO (~ 47–51 min) and the number of awakenings (~ 5.7–6.1) suggested mild-to-moderate fragmentation, estimated indirectly rather than with validated fragmentation indices; only time in bed (~ 8.1–8.2 h) remained within the expected range. Collectively, these findings suggest that sleep disturbances were not only persistent but also clinically relevant at both time points, consistent with the notion that stroke survivors may experience prolonged, rather than purely transient, sleep impairment during recovery [9, 17].

Interestingly, the functional profile of the sample suggests that sleep disturbances were not restricted to individuals with severe disability. Participants presented, on average, mild to moderate functional impairment, as indicated by median mRS scores, yet objective sleep parameters revealed marked alterations in sleep continuity and fragmentation. This finding suggests that sleep impairment is not solely a consequence of severe neurological deficits and may represent an intrinsic component of post-stroke recovery, with potential implications for daily functioning and overall quality of life [4, 16].

Several mechanisms may contribute to the persistence of sleep disturbances after stroke, including disruption of neural networks involved in sleep regulation, such as the thalamus, hypothalamus, and brainstem [16]. Circadian rhythm and rest–activity metrics, which can also be derived from actigraphy, were not analyzed in the present study and therefore cannot be discussed based on our data [19].

Sleep also plays a fundamental role in neuroplasticity and recovery after brain injury, influencing synaptic remodeling, memory consolidation, and immune regulation [20], and abnormal sleep duration has been associated with increased stroke risk and adverse cardiovascular outcomes [21]. Although our study did not directly measure neuroplasticity or cognitive outcomes, the persistence of poor sleep quality observed in our cohort underscores the potential for sleep disturbances to hinder optimal neurological recovery and daily functioning.

Another important observation in the present study was the considerable variability in sleep parameters across individuals. Although no statistically significant changes were detected at the group level, individual-level analysis revealed heterogeneous patterns of change in sleep efficiency, with an increase observed in 10 participants and a decrease in 8, consistent with the direction-only classification described in the Methods. Similar variability has been reported in previous studies of sleep disturbances after stroke [3, 17], and may reflect differences in stroke severity, lesion location, preexisting sleep disorders, or behavioral factors such as physical activity and daily routines during recovery. Future studies with larger, stratified samples may help identify predictors of these heterogeneous sleep-efficiency changes.

From a clinical perspective, the persistence of objectively measured sleep disturbances highlights the importance of systematic sleep assessment during post-stroke follow-up care, given its potential impact on cognitive recovery, mood regulation, and daily functioning. Current guidelines for post-stroke care increasingly emphasize the importance of addressing non-motor complications, including fatigue and sleep disturbances [22]. In this context, actigraphy is a feasible, non-invasive method for monitoring sleep–wake patterns in naturalistic environments and may help clinicians identify patients who could benefit from targeted sleep-related interventions [23].

The study has several strengths. First, seven-day actigraphy monitoring at each assessment enabled characterization of sleep patterns under real-world conditions, reducing bias from single-night measurements. Second, the longitudinal design objectively tracked sleep parameters from the subacute to later stages after stroke, providing repeated within-participant data that capture both group-level stability and individual-level variability in sleep parameters, in contrast to the largely cross-sectional or acute-phase focus of prior research. Finally, the inclusion of individuals with relatively mild functional impairment highlights that clinically relevant sleep disturbances are not limited to those with severe disability, adding nuance to the understanding of sleep problems across the spectrum of stroke recovery.

Some limitations should also be acknowledged. First, the sample was small (*n* = 18), heterogeneous, and predominantly composed of individuals with mild strokes (median NIHSS = 4) and preserved functional status (median mRS = 2) from a single Brazilian center; the findings are therefore exploratory and not generalizable to the broader stroke population, particularly to individuals with more severe impairment, cognitive deficits, or aphasia. Second, the considerable loss to follow-up (only 18 of 38 participants who completed baseline testing provided valid paired data; Fig. 1) may have introduced selection bias and further limits generalizability. Third, actigraphy cannot capture sleep architecture, arousals, circadian alignment, or sleep-disordered breathing, all clinically relevant in stroke and requiring polysomnography or dedicated circadian assessment; relatedly, sleep fragmentation was inferred from WASO and awakenings rather than validated fragmentation indices, which may underestimate true sleep instability. Fourth, the predominance of ischemic strokes (88.9%) precluded stratified analyses by subtype or lesion location, limiting mechanistic interpretation given the lesion-dependent role of structures such as the brainstem, thalamus, and hypothalamus in sleep–wake regulation. Fifth, data on sleep-affecting medications, rehabilitation exposure, and the association between sleep and the fatigue (FSS) and participation (SIS 3.0) measures collected were not analyzed, precluding adjustment for these confounders or exploration of functional correlates. Finally, the baseline window (1–3 months) and follow-up interval varied across participants (median 161 days, range 63–391); longer, more standardized follow-up is needed to characterize the long-term course of sleep disturbances after stroke.

In summary, objectively measured sleep parameters remained largely unchanged from the subacute phase to later stages of stroke recovery and were already outside normal reference ranges at both time points. These findings suggest that sleep disturbances may persist into the later stages of recovery; however, given the small, non-representative sample and the absence of statistically significant longitudinal change, longer-term studies with larger and more diverse cohorts are needed to determine whether these alterations reflect a persistent or chronic condition. These findings nonetheless reinforce the potential value of incorporating sleep assessment into post-stroke care.

## Conclusion

In this longitudinal study of a small, predominantly mild and functionally preserved sample of stroke survivors, objectively measured sleep parameters remained largely unchanged, but consistently outside normal reference ranges, from the subacute to later stages after stroke. These findings suggest that sleep disturbances may persist into the later stages of recovery; however, longer-term studies with larger and more representative samples are needed to determine whether these alterations reflect a persistent or chronic condition. These findings nonetheless reinforce the potential value of incorporating sleep assessment into post-stroke care.

## Data Availability

The datasets generated and/or analyzed during the current study are available from the corresponding author on reasonable request.
